# Collaborative Smartphone-Based User Positioning in a Multiple-User Context Using Wireless Technologies †

**DOI:** 10.3390/s20020405

**Published:** 2020-01-10

**Authors:** Viet-Cuong Ta, Trung-Kien Dao, Dominique Vaufreydaz, Eric Castelli

**Affiliations:** 1 Human Machine Interaction, University of Engineering and Technology, Vietnam National University, Hanoi 100000, Vietnam; cuongtv@vnu.edu.vn; 2 MICA Institute (HUST-Grenoble INP), Hanoi University of Science and Technology, Hanoi 100000, Vietnam; 3 University of Grenoble Alpes, CNRS, Inria, Grenoble INP, LIG, 38000 Grenoble, France; Dominique.Vaufreydaz@inria.fr (D.V.); Eric.Castelli@univ-grenoble-alpes.fr (E.C.)

**Keywords:** indoor localization, indoor navigation, multi-sensor fusion, multiple-user positioning

## Abstract

For the localization of multiple users, Bluetooth data from the smartphone is able to complement Wi-Fi-based methods with additional information, by providing an approximation of the relative distances between users. In practice, both positions provided by Wi-Fi data and relative distance provided by Bluetooth data are subject to a certain degree of noise due to the uncertainty of radio propagation in complex indoor environments. In this study, we propose and evaluate two approaches, namely Non-temporal and Temporal ones, of collaborative positioning to combine these two cohabiting technologies to improve the tracking performance. In the Non-temporal approach, our model establishes an error observation function in a specific interval of the Bluetooth and Wi-Fi output. It is then able to reduce the positioning error by looking for ways to minimize the error function. The Temporal approach employs an extended error model that takes into account the time component between users’ movements. For performance evaluation, several multi-user scenarios in an indoor environment are set up. Results show that for certain scenarios, the proposed approaches attain over 40% of improvement in terms of average accuracy.

## 1. Introduction

Satellite navigation systems such as GPS rely heavily on the exact timing of signals. Therefore, in an indoor environment where radio signals usually suffer from hazardous propagation effects, including fading, multipath, reflection, etc., their applicability is generally ignored. Among others, Wi-Fi and Bluetooth widely available in nowadays smartphones could be considered as low-cost alternative wireless-based solutions for positioning purpose. These two technologies come with the Receive Signal Strength (RSS) or Receive Signal Strength Index (RSSI) as a source of information from the environment. The information can be used in different approaches to compute the device’s position.

Novel Wi-Fi-based positioning methods on smartphones can find the position by scanning the available Wi-Fi access points in the surrounding environment. The mean distance error is around 5 m [1], due to the unreliable characteristics of the Wi-Fi signal propagation in an indoor environment. The Bluetooth characteristics are very similar to those of Wi-Fi in terms of the underlying physical radio characteristics and application aspect. Therefore, it is possible to develop a positioning system in an analogous manner to the Wi-Fi-based ones. However, the Bluetooth communication range is lower, which leads to a higher number of static beacons in deployment [2].

Nevertheless, in a multiple-user positioning context, the Bluetooth data can complement the Wi-Fi data to base a collaborative positioning framework [3]. When each user moves with a smartphone in a public area, it is possible to keep the smartphone’s Bluetooth in visible mode. Then, if a device sees another one nearby, the RSS from Bluetooth data can give an approximation of the relative range between the two devices. Given the estimated pair-to-pair distance information, it is possible to refine the output positions from Wi-Fi data. This approach does not require installing additional infrastructures and is compatible with the standard Bluetooth protocol, which is generally supported by smartphones.

There are several key challenges of the proposed approach. The first difficulty is the non-deterministic propagation characteristics of radio signals in an indoor environment. The noise affects both Wi-Fi output positions and Bluetooth output distances, especially in the case of moving users. The second one is the asynchronous events between the Wi-Fi and Bluetooth scanning processes of the smartphones. In other words, the scanning cycles for each technology in the smartphones are not guaranteed to start and finish at the same time. The time difference adds a level of uncertainty between the output positions from Wi-Fi data and Bluetooth data.

In this work, we try to overcome these problems by considering the so-called *Non-temporal* and *Temporal* approaches. In the former one, the distance error of the Wi-Fi output position is modeled by a Gaussian distribution. Similarly, another Gaussian distribution is used to describe the distance error between two devices from the Bluetooth inquiry process. Wi-Fi and Bluetooth position outputs within a short time interval are treated as if they happened in the same time window. An error function is then defined to measure the mismatch between the two distributions. By neutralizing the mismatch, it is possible to improve the position results of the Wi-Fi output. In the latter approach, the time component of the users’ movement is incorporated into the error function. The error function takes the positions of all the users as an input. We then make use of particle-filter-based tracking to minimize this error function. The observation model required by the particle filter is derived from a combination between Wi-Fi and Bluetooth scanning data.

This paper is an extension of a former conference article [4] with more updated results, detailed explanation and discussion; in addition, it is part of a Ph.D. thesis [5] and is arranged as follows. In Section 2, related works on smartphone-based indoor positioning and collaborative positioning are discussed. Section 3 introduces different techniques available for Wi-Fi positioning, then our proposed approaches for combining Wi-Fi and Bluetooth data are described in Section 4. Experiments and results are represented and discussed in Section 5, and finally, Section 6 contains conclusionary remarks and future prospects.

## 2. Related Works

Works on smartphone-based positioning in an indoor environment involve various kinds of technologies such as GPS, camera, Personal Dead Reckoning, Bluetooth, Wi-Fi, etc. Among them, Wi-Fi has been drawing the most attention thanks to its ubiquity, low cost and privacy. Popular approaches include geometry-based and fingerprinting-based ones. The geometry-based approaches rely on the estimation of certain geometry properties, including distance and angle, to calculate the device’s position. For calculating the distance, the well-known Log-Distance Path Loss (LDPL) model can be used [6]. More complex models are introduced in [7,8,9,10]. However, these models are defined with a set of parameters and usually require complex calibration processes.

Recently, the Wi-Fi fingerprinting-based methods have been preferred as they benefit from the ubiquity of the WLAN infrastructure. Recent works on smartphone-based Wi-Fi fingerprinting have reached a mean distance error of around 5 m [1,11]. In general, fingerprinting approaches include two phases: site survey and model training. In the first phase, a mapping from location to RSS/RSSI values in buildings is collected. The mapping is then used to learn the reverse relationship, from the scanned RSS/RSSI values to the locations. For learning techniques, the well-known K-Nearest Neighbors (KNN) and its alternatives are among the most popular ones [12,13,14]. In [15], the authors experiment with a wide range of KNN parameters to get a set of models. An ensemble result of the generated KNN models has a mean distance error of around 6 m. Besides KNN-based learning methods, decision tree-based learning methods can be used for learning the Wi-Fi signal characteristics with prominent results [4,16]. In [17], a comparison between a LDPL-based approach and fingerprinting-based approaches is introduced, showing that fingerprinting-based approaches have better performance. The accuracy of the fingerprinting-based approaches is not only dependent on the learning model but also on the quality of the mapping in the first phase. For the practicality of the system, the time and cost of the site-survey phase are important factors [18]. There are various works that aim to reduce the complexity of the site-survey phase in many aspects. In [19], the authors introduce a passive collection method that collects unlabeled fingerprints of the Wi-Fi signal. In the context of multiple users, Wi-Fi probes information can be used to build a survey-free fingerprinting database, as presented in [20]. As different devices often have different Wi-Fi signal characteristics, [21] proposes a Bayesian approach for dealing with device heterogeneity-related issues in indoor positioning.

Given the efficiency of the Wi-Fi-based positioning, the outputs from the system are often considered a data source for integrating with other positioning techniques. In [22], the authors build a distribution of possible points given a Wi-Fi measurement. The distribution is assumed to be Gaussian. The center is calculated from a fingerprinting approach. The distribution is then used to correct the output of an inertial-sensor-based system. A more complex fusion approach is introduced in [23], where the positions from the Wi-Fi system are used to shift the entire trajectory of PDR tracking by a gradient-descent-based search. In [24], a fusion model for Wi-Fi, PDR and Landmark is proposed in such a manner that the Wi-Fi RSS measurements are used to build a measurement model based on kernel density estimation.

For positioning, Bluetooth technology can be used in the same way as Wi-Fi. Bandara et al. [25] using up to four Bluetooth antennas as static stations to locate a Bluetooth tag within a room with an area of 4.5 m × 5.5 m. The RSSI value is used to classify the tag’s position between different subareas of the room. Pei et al. [26] employ a fingerprinting-based approach to track a moving phone. The setup includes only three Bluetooth beacons in a corridor-like space of 80 m long approximately. The horizontal error is reported as 5.1 m. More recent works employ the new Bluetooth Low Energy (BLE) technology [27]. BLE beacons are smaller, more energy efficient, and able to power up for a longer period, and hence are more convenient for positioning purposes. Faragher and Harle [2] provide an in-depth study of using BLE for indoor positioning. The distance error of their Bluetooth-based approach could reach as low as 2.6 m for 95% of the time. However, a high number of beacons should be deployed to reach the above performance. The study also addresses some issues of the BLE signal such as the scanning cycle, fast fading effects and Wi-Fi scanning interference. A similar performance for BLE-based indoor positioning is reported in [28], where the authors employ a fingerprinting-based approach with the RSS value from the installed BLE beacons.

In a scenario involving multiple devices, there are several works on collaborative localization. Those works rely on some specific wireless technologies that support peer-to-peer communication, including Bluetooth and Wi-Fi Direct, which are capable of discovering the existence of nearby neighbors. In [29], the task of detecting face-to-face proximity is studied. Smartphones are used to scan nearby visible Bluetooth devices in daily usage. From the received RSSI, the relative distance between two devices is calculated. The distance is then used to detect whether the two users are close to each other. To deal with noisy Bluetooth signals, additional techniques such as RSSI smoothing and light sensor data are introduced for calculating a more accurate distance. In [3], the Social-Loc system is proposed and uses Wi-Fi Direct technology for detecting two events, namely Encounter and Non-encounter, between each pair of users. In that work, the authors found the RSSI peak for separating Encounter and Non-Encounter events. These detected events are then used to improve the Wi-Fi fingerprinting and Dead Reckoning tracking. The drawback of Wi-Fi Direct technology is that it does not allow regular Wi-Fi scanning. Therefore, the proposed Social-Loc is more suitable for improving the Dead Reckoning tracking than the Wi-Fi fingerprinting tracking. In [30], a graph-based optimization process is used to combine different trajectories from multiple users. The graph is built by using the Personal Dead Reckoning data from the phone as moving constraints. It is then used to correct and extend the existing fingerprinting database.

## 3. Wi-Fi Fingerprinting for Indoor Positioning

In a large public area, fingerprinting-based approaches are often more preferable thanks to their lower labor cost in deployment.

In this section, different approaches of Wi-Fi fingerprinting methods for dealing with such types of training data are summarized. Based on the performance of these approaches, several ensemble strategies are proposed to improve the overall positioning performance. By combining uncorrelated models, it is expected to reduce the errors in cases of lacking training data. Alternative fingerprinting approaches rely on statistical models without directly dealing with the signal propagation complexity. The feature space is the RSS feature vector, and the target is the device’s position. Signal filtering can be added to boost the position accuracy.

Ensemble methods for improving learning models are a well-known approach in machine learning. For Wi-Fi fingerprinting-based positioning, Torres-Sospedra et al. in [15] propose an ensemble framework based on KNN models. The authors vary a set of parameters and then combine all the generated models to have a more stable performance. These parameters include the number K of considered neighbors, the distance/similarity measure of signal feature vector, the data representation, and the method of filtering out weak signals. However, because all the models are derived from the KNN model, their learning capability can be highly correlated. Thus, it may lead to an overfitting model when the ensemble step is carried out, especially in the context of limited training data. This study shares the similar idea of ensemble models but in a different way. Mostly, we aim to reduce the dependence on the KNN model by varying the Wi-Fi-signal-based features and the learning models.

In our approach, the Wi-Fi fingerprinting problem is first transformed into a standard learning problem. The learning problem includes a set of feature vectors and its desired labels. The label in this case is the coordinates of each sample. Two other feature sets, filter-based and hyperbolic-based features, are derived directly from the default raw signal features. The two additional features are reported with good performance for building Wi-Fi fingerprinting models. Three different learning models, which are KNN, Random Forest and Extreme Gradient Boost, can be used in the learning phase. The KNN model is well-known for working with Wi-Fi fingerprinting data. Both Random Forest and Extreme Gradient Boost models are tree-based methods that have good performance against various types of data. The two additional learning models are added to make the following ensemble step rely less on the KNN method. The learning targets are also selected between the regression and the classification objective functions.

### 3.1. Wi-Fi Fingerprinting Features

For building fingerprinting models, it is necessary to collect the training Wi-Fi signal data. Based on these data, signal characteristics are determined for each point. We can start with the raw features that only include the RSS value of nearby access points. The raw RSS value is expected to have a stable feature space for learning the target positions. Besides that, as our target is to vary learning spaces, two feature sets, the Filtering-based features [31] and the Hyperbolic Location Fingerprinting features [32], are added. Generally, the two latter feature sets could be considered as an improvement from the standard raw features. We add some minor modifications to make the added features applicable with the lack of training Wi-Fi RSS data.

#### 3.1.1. Raw Features

From the scanning process, the Wi-Fi access points in the surrounding environment are usually presented as pairs of MAC addresses and RSS values. The MAC address is used as the access point’s identifier, and the RSS value is the access point’s signal quality at the position of the mobile device. Each Wi-Fi data package within a short time window is grouped in the same scanning cycle. The raw features are built from a list of these two values of a scanning cycle. The length of a specific scanning cycle depends on the smartphone’s operating system. Practically, one could use a fixed time interval for defining a complete Wi-Fi scanning process of smartphones.

To create a matrix representation of the raw features, it is necessary to collect all the appeared access points within the collected data. With D access points, namely $AP_{1}$, $AP_{2}$,..., $AP_{D}$, the feature vector has exactly D dimensions. Assume at time t, there is a complete scan cycle at point P. Each scan cycle is then used to create a fixed-length feature vector r where each value $r_{i}$ is the RSS value of the access point $AP_{i}$ if it appears in the scan. In the other case, the $r_{i}$ takes the minimum value $MIN_{rss}$, which is selected to be lower than any other scanned RSS values that appear in the data. The selection of $MIN_{rss}$ could affect several types of learning models, such as KNN-based ones. More specifically, the KNN models use the distance metrics for comparing the similarity between two feature vectors. Therefore, the value of $MIN_{rss}$ can be selected to put more emphasis on the differences between the list of unseen access points from each feature vector.

#### 3.1.2. Filtering-Based Features

The filtering-based approach could be considered a direct improvement from the standard raw features. In the original version [31], Marques et al. split the whole feature space into sub-regions. The split criterion is the indices of the two strongest access points of the raw signal vector r. In a later study [33], the number of considered strongest access points was extended to three. Both works have good results when combined with KNN-based learning models. However, the results are tested in a large training data set (nearly 10,000 samples). The large training samples allow the filtering step to be able to split the feature space into sufficiently smaller subspaces. With smaller training data, its performance is moderate, as stated in [1].

In our context, to promote simplicity in deployment, suppose that only a small amount of data are available, then it is not a good option to adopt the original approach. Instead, the feature space could be split indirectly by adding features to the standard RSS feature vector. From the starting D dimensions, each corresponding to an appeared access point, an additional D features $s=\left(s_{1},s_{2},\ldots ,s_{D}\right)$ is added to the standard $r=\left(r_{1},r_{2},\ldots ,r_{D}\right)$. The value of $s_{i}$ is defined as:

(1) $$s_{i}=\{\begin{array}{ll} INF_{rss} & \;\mathrm{if}\;r_{i}\;\mathrm{is}\;\mathrm{one}\;\mathrm{of}\;\mathrm{two}\;\mathrm{highest}\;\mathrm{values}\;\mathrm{of}\;r,\\ 0 & \;\mathrm{otherwise}. \end{array}$$

Then, the new feature vector q is a combination of r and s:

(2) $$q=\left(r_{1},r_{2},\ldots ,r_{D},s_{1},s_{2},\ldots ,s_{D}\right).$$

The purpose of using $INF_{rss}$ instead of a simple value constant 1 is to allow the combined vector q to work with several types of distance metrics. By selecting an appropriate value of $INF_{rss}$, it is possible to put more weight into the difference in s rather than the difference in r.

The resulting vector length is double the standard one’s. The number of strongest access points can be selected as an adjustable parameter. Normally, a value of 2 or 3 is chosen for effective splitting of the standard RSS feature spaces. In comparison to the original filter version, there is a minor modification in our implementation version.

#### 3.1.3. Hyperbolic Location Fingerprinting Features

As the RSS value is highly dependent on the device, several methods have been proposed to overcome the hardware-dependent issue, including mean normalization, application of linear transformations, and usage of signal-calibration free approaches. Roughly speaking, most methods rely on a linear correlation of RSS values for different devices. The calibration-free approaches can be considered more general as they do not need to additionally calibrate data to find the linear transform. The methods encode the transforms’ parameters directly into the feature vector. Popular methods include the RSS difference [34] and hyperbolic location fingerprinting (HLF) features [32].

For reducing the effect of noisy RSS value between different devices, the HLF takes the relative difference for every pair of access points. Other than that, the RSS values is transformed into logarithm space. More specifically, from the standard raw features r with D dimensions, the HLF is a vector h which has a size of D(D−1)/2. The explicit values of h are given as:

(3) $$h=\left(\mathrm{diff}(r_{1},\;r_{2}),\ldots ,\mathrm{diff}(r_{1},\;r_{D}),\mathrm{diff}\left(r_{2},\;r_{3}\right),\ldots ,\mathrm{diff}\left(r_{2},\;r_{D}\right),\ldots ,\mathrm{diff}\left(r_{D-1},r_{D}\right)\right),$$

where the different operations between two RSS values $r_{i}$ and $r_{j}$ is given by:

(4) $$\mathrm{diff}\left(r_{i},\;r_{j}\right)=\log\frac{r_{i}}{r_{j}}-\;\log\frac{1}{r_{\max}},$$

with $r_{\max}$ being the maximum RSS value.

When there are hundreds of access points available in the environment, the transformation in Equation (3) results in thousands of dimensions in the feature spaces. Thus, collecting enough data for training the HLF features could be problematic. Therefore, applying a further dimension reduction operator to get a suitable feature representation from the generated HLF features could be suitable to reduce the dimensions from D(D−1)/2 to $D^{\prime }$. Several possible approaches are Random Tree Embedding [35], Principle Component Analysis, and Truncated-Single Value Decomposition [36]. The value of $D^{\prime }$ should be chosen as small to make the learning process in later training phases feasible.

After the feature selection step, the collected data can be represented as the lists of pairs:

(5) $$S=\left(<X^{1},P^{1}>,<X^{2},P^{2}>,\ldots ,<X^{M},P^{M}>\right),$$

with $X^{i}$ being the correspondent N-dimensional vector and $P^{i}$ the position of the $i^{th}$ sample for i∈[1,M]. For simplicity, the notation $X^{i}$ could be in one of three types of features, the raw features r, the filters-based features s, or the HLF-based features h.

### 3.2. Learning Models

For learning the mapping from RSS-based vector to position, a number of model families are available for usage, which include Nearest Neighbors, Random Forest [37] and Extreme Gradient Boost [38]. While the KNN model is a popular choice to work with the Wi-Fi fingerprinting data, the others are tree based. They are capable of learning on a small amount of training data. Each type of model can also be applied with either options, i.e., classifier or regressor.

#### 3.2.1. K-Nearest-Neighbor Model

The K-Nearest Neighbors (KNN) model requires a distance function between two vectors in the feature space. Similar distance errors could be achieved with a group of distance functions including cosine, Euclidean, Hellinger, and Chi-square. We choose the Euclidean function for measuring the distance between two feature vectors. For an input $X^{new}$, the standard KNN algorithm finds K samples in S, which have the smallest distances to $X^{new}$. Assume the list of K samples is:

(6) $$\mathrm{nearest}\left(X^{new},d^{euclide}\right)=\left(<X^{u_{1}},Y^{u_{1}}>,<X^{u_{2}},Y^{u_{2}}>,\ldots ,<X^{u_{K}},Y^{u_{K}}>\right),$$

then the corresponding $P^{new}$ is computed as the centroid of all $P^{u_{i}}$ in the above list. Parameter K defines how many nearest samples in the feature space are taken for computing $P^{new}$. A small value of K tends to make to model overfitting. For example, with K=1, $P^{new}$ is the position with the nearest sample in the training set S. When K increases, more nearest samples are taken into the computation, thus making the output more robust. However, if there are only a few samples, $P^{new}$ may fluctuate between them. In the specific task of Wi-Fi fingerprinting, K is usually chosen from in the range of [1, 10].

There are some variants of the KNN model. The most popular one is the weighted-KNN approach, in which $P^{new}$ is calculated by a weighted sum of average of $P^{u_{j}}$. The specific weight is a function, which is computed from the similarity between $X^{new}$ and $X^{u_{j}}$.

#### 3.2.2. Random Forest Model

Random Forest (RF) model is based on building a list of decision trees. Each tree would split the feature space into random subspaces. Each subspace is expected to contain the samples that have identical or similar target positions. The work in [39] shows that the RF model can be justified as an adaptive weighted-KNN model.

**Figure 1:**
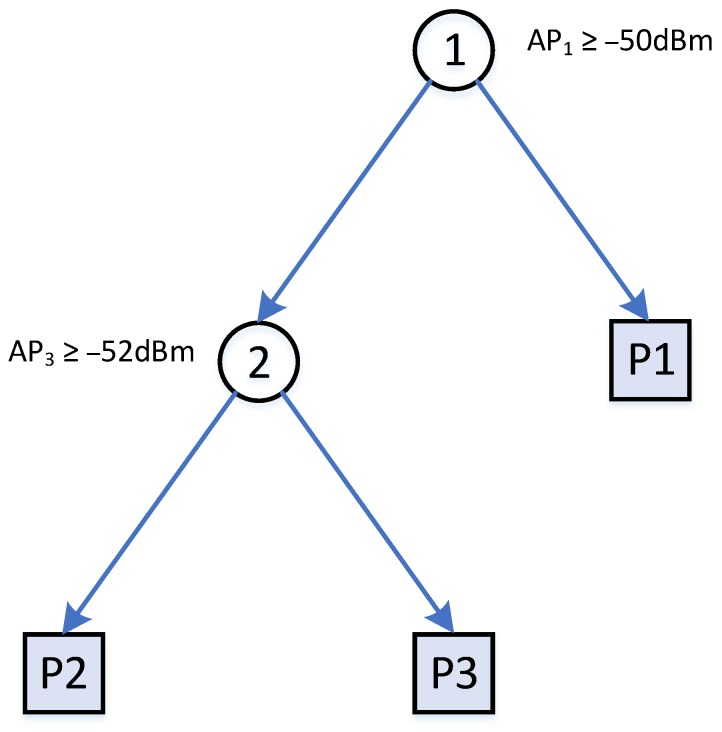
A decision tree with two internal and three leaf nodes for splitting $X_{1}$, $X_{2}$, $X_{3}$ in Table 1.

For training a standard decision tree, the training data S is split from top down by a pair of feature and values until a certain criterion is met. The criterion is based on the depth of the tree or errors within for defining a leaf node. Table 1 shows a set of three training samples on the raw RSS data. A decision tree could be built as shown in Figure 1. In this example, the building process stops when every leaf contains only one sample. There are also other decision trees with different node splitting criteria that can split the tree samples $X_{1},X_{2}$ and $X_{3}$.

Given a training data set, there are several well-known methods for building a decision tree such as ID3 [40] or CART [41]. Because the building decision tree process always seeks for an optimal way to split the feature space, the resultant tree is likely overfitting. The RF model is introduced for overcoming the overfitting issue by building a set of decision trees. In general, none of the trees are optimal, but all have a predictive capability. Bagging methods and a random subset of features are employed for lowering the correlation between each tree in the forest. The output of the RF model is the mean of all the predictions of each decision tree in the forest.

#### 3.2.3. Extreme Gradient Boost Model

Extreme Gradient Boost (XGB) model is also a tree-based learning method. The training process includes several rounds. Starting from a simple prediction, trees are added to the model based on a boosting approach, which tries to reduce the error of the objective function of the previous step. Popular objective functions for the XGB model include the mean square error for regression or logistic function for classification. Apart from the objective function, additional components that measure the model complexity are also added. The objective function is chosen in a way that it can compute the gradient based on features values. Therefore, at each splitting step, the built tree can choose the right ways to split the feature space. The model is also included with other techniques for preventing overfitting such as a random subset of features or early stopping criteria.

## 4. Using Bluetooth Data to Improve Wi-Fi Positioning

Wi-Fi and Bluetooth are two data streams which provide different information that can contribute to the derivation of the users’ position. In the case of multiple users, the distance from one user to others can be calculated from the Bluetooth scanning process. A way to combine the two different data streams is to use a central server that keeps all the available positioning information from each participating device. More precisely, the information includes the estimated positions from Wi-Fi data and the estimated distances between pairs of devices from Bluetooth data.

### 4.1. Fusion Framework for Wi-Fi and Bluetooth

For the fusion, Wi-Fi and Bluetooth data are sent to the server. For each completed Wi-Fi scan cycle, the device sends its identifiers (MAC addresses) of the found access points and the corresponding RSS values. Each Wi-Fi scan cycle takes a few seconds and is dependent on the device itself as well as the number of access points. The second type is the Bluetooth scanned information, which also includes the Bluetooth MAC addresses of visible devices and the corresponding RSS values. On the server, the data from Wi-Fi and Bluetooth scans give different ways to calculate the users’ positions.

**Figure 2:**
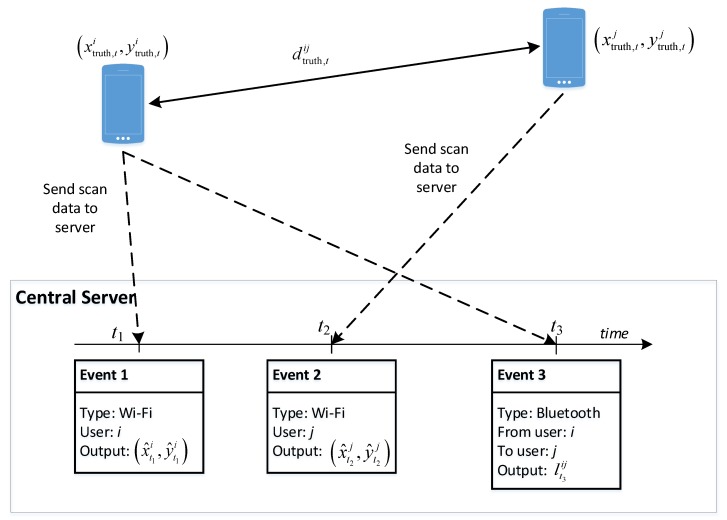
Data sent from two devices to the central server and derived information at the server for estimating each devices’ position.

Figure 2 illustrates the principle of our approach with an example context that involves two users, namely i and j. The two users’ devices keep gathering information on Wi-Fi access points and Bluetooth devices in the environment and sending them to the server. Once the server receives the data, it will store it as events associated with either Wi-Fi or Bluetooth typed labels. The figure illustrates three events: two Wi-Fi scans and a Bluetooth scan. Assume that the real positions of users i and j at time t are denoted as $\left(x_{\mathrm{truth},t}^{i},y_{\mathrm{truth},t}^{i}\right)$ and $\left(x_{\mathrm{truth},t}^{j},y_{\mathrm{truth},t}^{j}\right)$, respectively, the real distance between the two users is denoted as $d_{\mathrm{truth},t}^{ij}$.

At time $t_{1}$, the position of user i can be estimated from the Wi-Fi scan information device i, and is denoted as $\left({\hat{x}}_{t_{1}}^{i},{\hat{y}}_{t_{1}}^{i}\right)$. Similarly, at time $t_{2}$, when the Wi-Fi information from device j is available when it completes a scan, we can estimate the position of user j, which results in $\left({\hat{x}}_{t_{2}}^{i},{\hat{y}}_{t_{2}}^{i}\right)$. Besides that, the Bluetooth scan information when available can lead to an estimated relative distance between these two users. At time $t_{3}$, if device i is found by device j with its Bluetooth scan process, we can also make an estimation $l_{t_{3}}^{ij}$ of the relative distance real distance $d_{\mathrm{truth},t_{3}}^{ij}$, based on the corresponding RSS value.

Alternatively, we can estimate $d_{\mathrm{truth},t_{3}}^{ij}$ differently by using the output position from the Wi-Fi information for the two users. As the true value $d_{\mathrm{truth},t_{3}}^{ij}$ is unknown, our task is looking for ways to minimize the difference between the values estimated independently from the two sources. However, this Wi-Fi information is generally not available in a synchronous manner with the Bluetooth information. In the current example, it is available at times $t_{1}$ and $t_{2}$, but not $t_{3}$. To address the asynchronous fusion of the relationship between Wi-Fi and Bluetooth for improving positioning results, we propose two approaches that utilize the timestamp differently:

- The first approach is called *Non-temporal*, where the temporal relationship between events which are within a time interval window is ignored. They are treated as if they happened at the same time. An error estimation function is established by using the users’ position as the function’s parameters. By minimizing the error function, it can be used to smooth the mismatch between Wi-Fi data and Bluetooth data, thus reducing the positioning error from Wi-Fi.
- The second approach is called a *Temporal* one, in which we introduce the time component into the basic error function of the first approach. More precisely, the new error function includes the devices’ position at each timestamp. For minimizing the new error function, a particle-based approximation is carried out.

For both approaches, in principle, any Wi-Fi and Bluetooth models that are able to deduce the relative user distance can be used. In this study, a fingerprinting model is used for Wi-Fi, and the LDPL model for Bluetooth.

### 4.2. Non-Temporal Approach

In the *Non-temporal* approach, a sliding window of size Δt seconds is used. All the events from t to t+Δt are considered as if they happened at the same time. Given a pair of users i and j, one can assume that there exists both Wi-Fi data and Bluetooth data within the time frame from t to t+Δt. Let $w^{i}$ and $w^{j}$ be the Wi-Fi scan information from the two users, and $rss^{ij}$ be the RSS value of the Bluetooth scan. In this *Non-temporal* approach, we remove the time variable t from the parameters. The likelihood function with the two users’ positions $\left(x^{i},y^{i}\right)$ and $\left(x^{j},y^{j}\right)$, and the parameters $P\left(x^{i},y^{i},x^{j},y^{j}|w^{i},w^{j},rss^{ij}\right)$ are created by decomposing the function into three separate components:

(7) $$P\left(x^{i},y^{i},x^{j},y^{j}|w^{i},w^{j},rss^{ij}\right)=P_{W}\left(x^{i},y^{i}|w^{i}\right)\times P_{W}\left(x^{j},y^{j}|w^{j}\right)\times P_{B}\left(x^{i},y^{i},x^{j},y^{j}|rss^{ij}\right),$$

where $P_{W}\left(x^{i},y^{i}|w^{i}\right)$ and $P_{W}\left(x^{j},y^{j}|w^{j}\right)$ are the error estimations from Wi-Fi data of users i and j, respectively, and $P_{B}\left(x^{i},y^{i},x^{j},y^{j}|rss^{ij}\right)$ is the error from the Bluetooth data.

Let $\left({\hat{x}}^{i},{\hat{y}}^{i}\right)$ be the computed position from the scan $w^{i}$. By assuming that the distribution of the real position $\left(x^{i},y^{i}\right)$ is a 2D Gaussian around the estimated position $\left({\hat{x}}^{i},{\hat{y}}^{i}\right)$, then $P_{W}\left(x^{i},y^{i}|w^{i}\right)$ is determined by:

(8) $$P_{W}\left(x^{i},y^{i}|w^{i}\right)=\frac{1}{\sqrt{2\pi }\delta_{w}}e^{-\frac{{\left(x^{i}-{\hat{x}}^{i}\right)}^{2}+{\left(y^{i}-{\hat{y}}^{i}\right)}^{2}}{2\delta_{w}^{2}}}.$$

Similarly, $P_{W}\left(x^{j},y^{j}|w^{j}\right)$ can also be determined given the estimated position $\left({\hat{x}}^{j},{\hat{y}}^{j}\right)$ from the Wi-Fi positioning model:

(9) $$P_{W}\left(x^{j},y^{j}|w^{j}\right)=\frac{1}{\sqrt{2\pi }\delta_{w}}e^{-\frac{{\left(x^{j}-{\hat{x}}^{j}\right)}^{2}+{\left(y^{j}-{\hat{y}}^{j}\right)}^{2}}{2\delta_{w}^{2}}}.$$

To estimate the Bluetooth part of the estimated likelihood, we first calculate $l^{ij}$ from $rss^{ij}$ by using the LDPL model:

(10) $$l^{ij}=l_{0}\times 10^{\frac{rss^{ij}-rss_{l_{0}}}{10n}}.$$

where $rss_{l_{0}}$ is the RSS value at distance $l_{0}$, and n is the path-loss exponent. The three values $rss_{l_{0}}$, n, and $l_{0}$ are known constant parameters. The value of $l^{ij}$ is the estimation of the real distance $d^{ij}$, which comes directly from the real positions of users i and j, that are $\left(x^{i},y^{i}\right)$ and $\left(x^{j},y^{j}\right)$:

(11) $$d^{ij}=\sqrt{{\left(x^{i}-x^{j}\right)}^{2}-{\left(y^{i}-y^{j}\right)}^{2}}.$$

One can assume that $l^{ij}$ has a Gaussian distribution around $d^{ij}$, then the Bluetooth likelihood can be estimated by another Gaussian kernel:

(12) $$P_{B}\left(x^{i},y^{i},x^{j},y^{j}|rss^{ij}\right)=\frac{1}{\sqrt{2\pi }\delta_{b}}e^{-\frac{{\left(d^{ij}-l^{ij}\right)}^{2}}{2\delta_{b}^{2}}},$$

where $\delta_{b}$ is a constant characterizing the reliability of the LDPL on the RSS Bluetooth signal.

From Equations (8)–(10), the likelihood function in Equation (7) can be rewritten as:

(13) $$P\left(x^{i},y^{i},x^{j},y^{j}|w^{i},w^{j},rss^{ij}\right)=C\times e^{-g},$$

where C is a constant, and g is a function of $x^{i},\;y^{i},\;x^{j},\;y^{j}$, that is:

(14) $$g=\frac{{\left(x^{i}-{\hat{x}}^{i}\right)}^{2}+{\left(y^{i}-{\hat{y}}^{i}\right)}^{2}+{\left(x^{j}-{\hat{x}}^{j}\right)}^{2}+{\left(y^{j}-{\hat{y}}^{j}\right)}^{2}}{2\delta_{w}^{2}}+\frac{{\left(\sqrt{{\left(x^{i}-x^{j}\right)}^{2}-{\left(y^{i}-y^{j}\right)}^{2}}-l^{ij}\right)}^{2}}{2\delta_{b}^{2}}.$$

**Figure 3:**
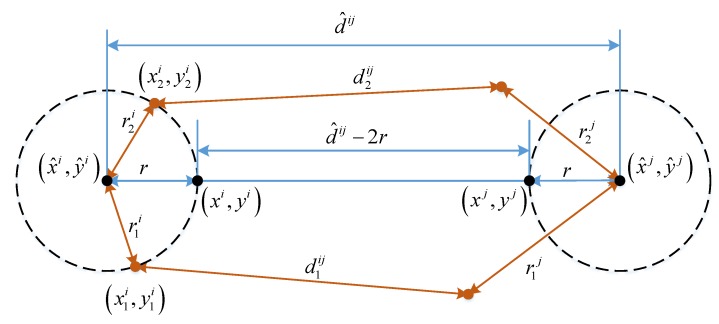
Several ways for evaluating *g* by using its symmetric property.

Figure 3 illustrates of the symmetric properties g. The pair of positions $\left(x^{i},y^{i}\right)$ and $\left(x^{j},y^{j}\right)$ can be used for finding the minimum value of $g\left(x^{i},y^{i},x^{j},y^{j}\right)$. It also shows the symmetric property of g. The value of $g\left(x_{1}^{i},y_{1}^{i},x_{1}^{j},y_{1}^{j}\right)$ is equal to $g\left(x_{2}^{i},y_{2}^{i},x_{2}^{j},y_{2}^{j}\right)$ if $r_{1}^{i}$ is equal to $r_{2}^{i}$ and $r_{1}^{j}$ is equal to $r_{2}^{j}$. For a fast computation of the minimum value of g, two constraints can be used based on its symmetric property. The first constraint is that the four points $\left(x^{i},y^{i}\right)$, $\left(x^{j},y^{j}\right)$, $\left({\hat{x}}^{i},{\hat{y}}^{i}\right)$, and $\left({\hat{x}}^{j},{\hat{y}}^{j}\right)$ are aligned. The second constraint is that the distance between $\left(x^{i},y^{i}\right)$ and $\left({\hat{x}}^{i},{\hat{y}}^{i}\right)$ and the distance between $\left(x^{j},y^{j}\right)$ and $\left({\hat{x}}^{j},{\hat{y}}^{j}\right)$ are equal.

The function is then rewritten as a function of the distance r between $\left(x^{i},y^{i}\right)$ and $\left({\hat{x}}^{i},{\hat{y}}^{i}\right)$, whose minimum value can be easily computed. The updated positions of users i and j are then calculated from r by using the two mentioned constraints:

(15) $$g\left(r\right)=\frac{r^{2}}{\delta_{w}^{2}}+\frac{{\left({\hat{d}}^{ij}-2r-l^{ij}\right)}^{2}}{2\delta_{b}^{2}}.$$

The time interval of validity for the incoming Wi-Fi and Bluetooth data makes the error probability able to be approximated by function g. However, it introduces several drawbacks. The first one is due to the users’ movement, i.e., the distances estimated using Wi-Fi and Bluetooth data may vary in different time windows. The second drawback is the difficulty to determine the time interval length Δt for grouping consecutive Wi-Fi and Bluetooth data. The later *Temporal* approach is designed to address these problems of the *Non-temporal* approach.

### 4.3. Temporal Approach

In the *Temporal* approach, we attempt to use the temporal relationship in the likelihood function P. Instead of relying only on the position of two users at specific timestamps to measure the errors; the likelihood function is extended to include the moving path of all the users. Each moving path is considered as a sequence of points. The new likelihood function would take all the points as parameters.

We first construct the likelihood function F, which is a more complete form of P that is based on three probability functions. A motion model M is used to establish the relationship between the positions at time indices τ and τ+1 when a user moves. Two functions W and B are defined as the probability distributions of the Wi-Fi scan results and the distance based on Bluetooth RSS values from each pair of devices, respectively.

Assume that there are N users to track in T time indices. The time component is added to the position of user i at time index τ as $\left(x_{\tau }^{i},y_{\tau }^{i}\right)$. Normally, we can select the time difference value based on the specific purpose of the positioning system. For modeling purposes, it is required that the time index τ contains all the event timestamps from Wi-Fi and Bluetooth of all the participant devices. Approximately, all the float-typed timestamps could be rounded to the nearest integer values. The motion model for each user i is defined as a probability function characterizing the dependency of the current position to the previous one, or $M\left(x_{t},y_{t}|x_{t-1},y_{t-1}\right)$. The moving component for T time indices for each user i is then calculated by:

(16) $$F_{i}^{M}=\overset{T}{\underset{\tau =1}{\prod }}M\left(x_{\tau }^{i},y_{\tau }^{i}|x_{\tau -1}^{i},y_{\tau -1}^{i}\right).$$

For each user i, assume that K within T time indices corresponds to the Wi-Fi scan records. Denote the timestamps for Wi-Fi events as $u_{1}$, $u_{2}$,..., $u_{K}$, then for each $u_{k}$, the corresponding Wi-Fi scan information is denoted as $w_{u_{k}}^{i}$. The function W is used to estimate the likelihood probability $W\left(x_{u_{k}}^{i},y_{u_{k}}^{i}|w_{u_{k}}^{i}\right)$. Then, the Wi-Fi component $F_{i}^{W}$ of user i is established from all the K available Wi-Fi scans:

(17) $$F_{i}^{W}=\overset{K}{\underset{k=1}{\prod }}W\left(x_{u_{k}}^{i},y_{u_{k}}^{i}|w_{u_{k}}^{i}\right).$$

The Bluetooth evolves for a pair of users i and j. Suppose that there are L Bluetooth data that arrive at timestamps $v_{1}$, $v_{2}$,..., $v_{L}$, it is possible to chain the error function over the L timestamps as follows:

(18) $$F_{ij}^{B}=\overset{L}{\underset{l=1}{\prod }}B\left(x_{v_{l}}^{i},y_{v_{l}}^{i},x_{v_{l}}^{j},y_{v_{l}}^{j}|rss_{v_{l}}^{ij}\right).$$

By joining the three functions, the total likelihood function F could be rewritten as:

(19) $$F=\left(\overset{N}{\underset{i=1}{\prod }}F_{i}^{M}\right)\left(\overset{N}{\underset{i=1}{\prod }}F_{i}^{W}\right)\left(\overset{N}{\underset{i=1}{\prod }}\overset{N}{\underset{j=1}{\prod }}F_{ij}^{B}\right).$$

At this step, one could select the explicit form of M, W and B and process them to find the maximum value of F. The number of parameters in F needing to be determined totally depends on the number of users and the tracking time. As F includes a motion model M, particle-filter-based approximation is a natural way to find the maximum value of F. In addition, the particle filter process would be more flexible than writing down W and B in explicit forms.

#### 4.3.1. Motion Model

**Figure 4:**
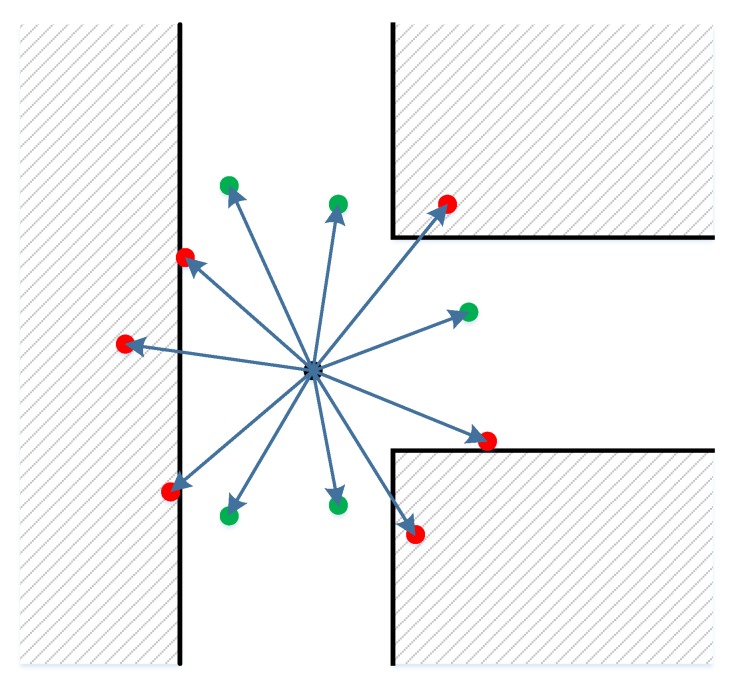
A simple moving model with the black dot being the initial particle. The walls are used as a constraint to remove bad particles.

For user i at time t, there is a set $S_{i}^{t}$ of particles, which represents the position probability distribution. For motion model M, without additional information from inertial sensors, the movement of the user could take random values as moving speed v and heading direction h. While there is no constraint on the value of h, the moving speed v should be suitable with a typical indoor movement. In our specific implementation, we generate the moving speed based on a normal distribution around a mean speed value. This mean value is determined statistically according to the walking action in an indoor environment. The heading is generated with a uniform distribution in the range [0, 2π]. An additional wall-cross checking step is added for removing bad particles. Figure 4 depicts an example of the motion model M for generating new particles. The black center dot is the initial particle. The wall boundaries are represented with black lines. New particles are then generated with a normally distributed moving speed around the mean value and a uniformly distributed heading direction. The green particles are valid and kept. The red ones, in contrast, which lie within the walls, are bad and removed.

#### 4.3.2. Observation Model

With the particle-filter-based approximation, the likelihoods $F^{W}$ and $F^{B}$ given by Wi-Fi and Bluetooth components can be transformed into an observation model. The score for a specific particle $p_{i}^{t}\in S_{i}^{t}$ is calculated by:

(20) $$score\left(p_{i}^{t}\right)=score_{W}\left(p_{i}^{t}\right)+score_{B}\left(p_{i}^{t}\right),$$

where $score_{W}\left(p_{i}^{t}\right)$ and $score_{B}\left(p_{i}^{t}\right)$ are the Wi-Fi and Bluetooth components, respectively.

#### 4.3.3. Wi-Fi Scoring Component Based on Classification

If there exists a Wi-Fi scan w at time t, then $score_{W}\left(p_{i}^{t}\right)$ is computed by using a local estimation of the probability output of the Wi-Fi fingerprinting model. Normally, $score_{W}\left(p_{i}^{t}\right)$ can be simply computed by assuming that the error distribution is 2D Gaussian with pre-defined standard deviation like in Equation (8). However, the estimation is heavily dependent on the accuracy of the Wi-Fi fingerprinting model. In addition, when the particles are close to each other, the score is nearly identical, which makes the selection step become difficult.

**Figure 5:**
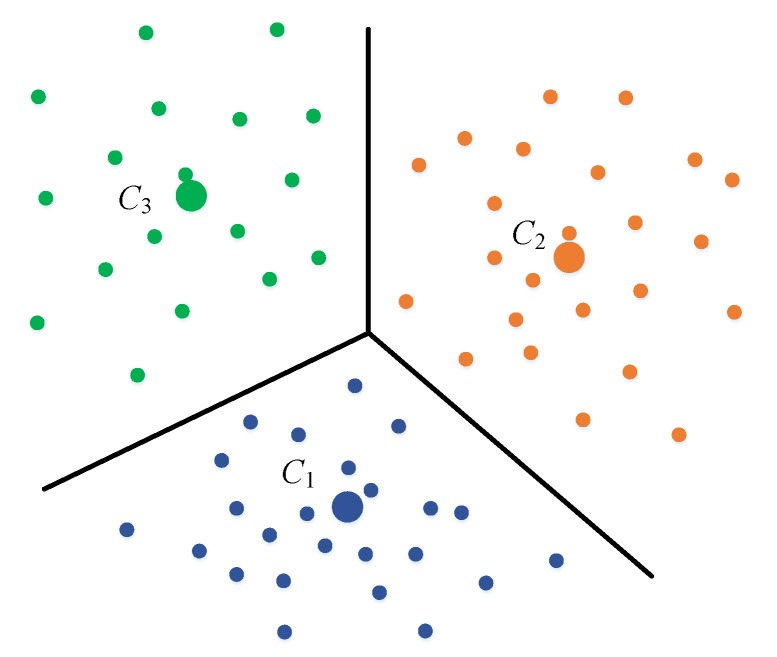
K-means clustering process used to group nearby training points into separated clusters, and position targets transformed from coordinates into labels.

To remove the strong effect of a single output position and make the score more conservative, a local-based scoring system is used in our approach. The area is first divided into D separated smaller ones whose centers are $C_{1}$, $C_{2}$,..., $C_{D}$. To compute the coordinates of the centers, a K-means clustering approach is used on all the appeared positions using the available fingerprinting data. Nearby positions are grouped into separated clusters. The center position of each cluster is the average of all the points that belong to each group. Figure 5 illustrates three centers with their boundaries. The boundaries are then used to transform the positions into appropriate indices. A 2D point (x,y) can be assigned to label d if the center $C_{d}$ is the nearest center to (x,y). With this mapping, it is possible to transform the Wi-Fi fingerprinting model from a standard regression problem into a classification problem [16]. Assume the training samples in the fingerprinting database have the form 〈rss,P〉 where rss is the RSS vector and P is the coordinates where the RSS is collected. The target value P can be mapped to one of the labels from 1 to D, by assigning P to the nearest center among $C_{1}$, $C_{2}$,..., $C_{D}$, as shown in Figure 5.

In the prediction step, let $prob_{w}=\left\{a_{1},a_{2},\ldots ,a_{D}\right\}$ be the probability of the predicted output of w belonging to the clusters, then $score_{W}\left(p_{i}^{t}\right)$ can be computed as:

(21) $$score_{W}\left(p_{i}^{t}\right)\;=\overset{D}{\underset{i=1}{\sum }}score_{C_{i}}\left(p\right),$$

where $score_{C_{i}}\left(p\right)$ is a scaled-value from $prob_{w}$, with respect to the maximum distance $d_{C_{i}}^{\max}$ and minimum distance $d_{C_{i}}^{\min}$, corresponding to all the available particles:

(22) $$score_{C_{i}}\left(p\right)\;=a_{i}\times \left(1-\frac{d\left(p,C_{i}\right)-d_{C_{i}}^{\min}}{d_{C_{i}}^{\max}-d_{C_{i}}^{\min}}\right).$$

**Figure 6:**
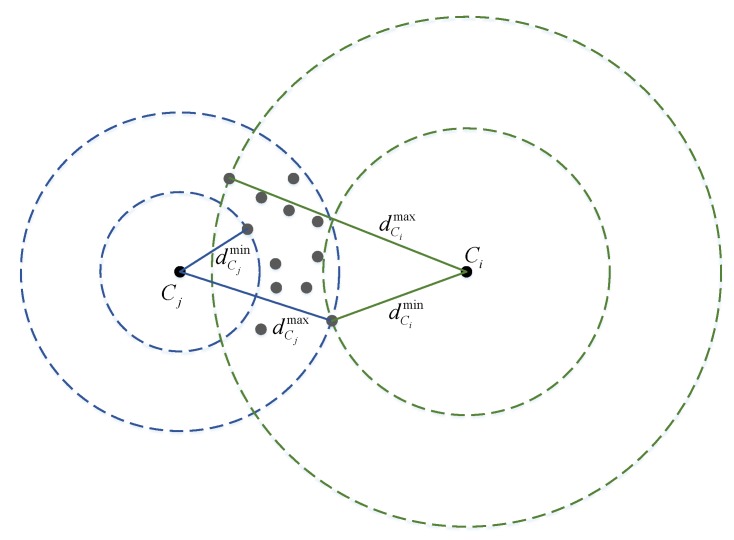
Scoring function for particles (gray dots) with two centers $C_{i}$ and $C_{j}$. The maximum and minimum distances are used to scale the observation probability.

A constant parameter $\Delta_{1}t$ defines the effective window length for a Wi-Fi scan. This mechanism is illustrated in Figure 6.

#### 4.3.4. Observation Model from Bluetooth

Similarly, $score_{B}\left(p_{i}^{t}\right)$ can be calculated if there is a Bluetooth scan involving user i around time t. Without a loss of generality, we assume that the available Bluetooth scan is $rss_{ij}^{t}$, which specifies the RSS value of device i measured by device j. The update rule for $score_{B}\left(p_{i}^{t}\right)$ is defined as:

(23) $$score_{B}\left(p_{i}^{t}\right)=\underset{p_{j,k}^{t}\in S_{j}^{t}}{\sum }score_{W}\left(p_{j,k}^{t}\right)\times B\left(p_{i}^{t},p_{j,k}^{t}|rss_{ij}^{t}\right),$$

where the subscript k indicates the need to calculate repeatedly for each $p_{j,k}\in S_{j}^{t}$. The likelihood $B\left(p_{i}^{t},p_{j,k}^{t}|rss_{ij}^{t}\right)$ is computed by using a similar process as the computation of the likelihood $P_{B}$ in the *Non-temporal* approach. Using Equation (12), the likelihood B can be rewritten as a function of distance d between two particles $p_{i}^{t}$ and $p_{j,k}^{t}$ and distance l derived from $rss_{ij}$ using the LDPL model. A constant parameter $\Delta_{2}t$ is also added to define the effective interval length for a Bluetooth scan.

## 5. Experiment and Results

### 5.1. Data Collection

**Figure 7:**
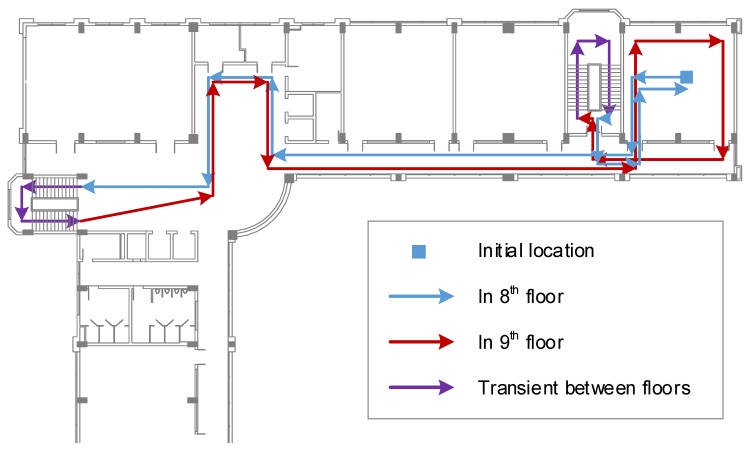
Moving path and checkpoints in two floors.

To evaluate the performance of the two proposed approaches, an experiment in an office environment that includes two floors is set up. All recordings follow one common trajectory, which is composed of the corridors, office rooms and stairs located in two consecutive floors. This trajectory is defined by several checkpoints. The path is illustrated in Figure 7. The length of the path is approximately 200 m, which usually takes about 300 s of walking at an average speed.

Different scenarios are designed in such a way that the additional Bluetooth scanning data can provide useful information for smoothing the Wi-Fi positioning output. Each of them involves groups of two to four users. The users were instructed to carry the devices and move along the designed path. While all the users walk along the same path, their relative distances are variable. In the recordings, we use a time interval of 0.5 s for tracking the users. Each time a checkpoint is reached, the time is recorded. As the checkpoint coordinates and their reaching timestamps are known, they can later be used to interpolate the ground truth for training and evaluation.

We selected four devices including two smartphones and two tablets for the positioning scenario (see Table 2). Each device is programmed to scan Wi-Fi access points and available Bluetooth devices in the environment. All of them run Android and use the same application for collecting Wi-Fi and Bluetooth data.

### 5.2. Bluetooth Model for Distance Deduction

In the first part, the model parameters for estimating the distance from RSS value need to be determined. From Equation (10), it is mandatory to find the appropriate $l_{0}$ and corresponding RSS value at $l_{0}$, namely $rss_{l_{0}}$. In this experiment, we take the path loss exponent n=1.8 as the default parameter for the indoor office, as suggested by [6].

**Figure 8:**
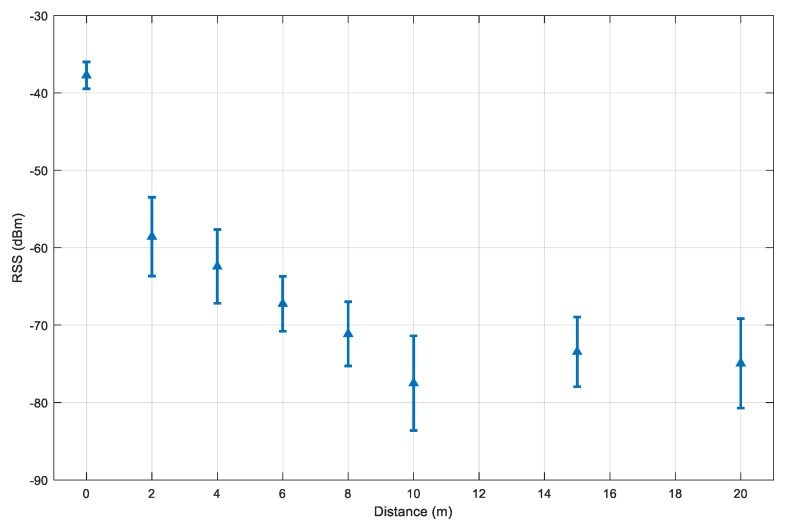
The mean RSS and its standard deviation values for selected distances between two smartphones.

A test device is set to scan the other visible Bluetooth devices that are set up so that there is no obstacle between the two ends, thus, the effect of non-line-of-sight propagation is ignored. For each distance, the collection period is set to 300 s. With the devices in use, the Bluetooth inquiry cycle takes around 12 s on average. This results in around 25 to 30 samples per distance. However, when the distance is too large, the number of collected samples reduces. At the distance of 20 m, there are only 10 inquiry samples. Figure 8 illustrates the experiment results.

The distance l of 0 m has the highest RSS value and the lowest standard deviation. The decrement pattern becomes more stable for the distance from 2 m to 10 m. Beyond the range of 10 m, the RSS value starts to be less predictable and packages are dropped. This explains why the mean distances at 15 m and 20 m are higher than at the 10 m. With this consideration, we selected $l_{0}=2\;m$ and $rss_{l_{0\;}}=-58\;\mathrm{dBm}$.

**Figure 9:**
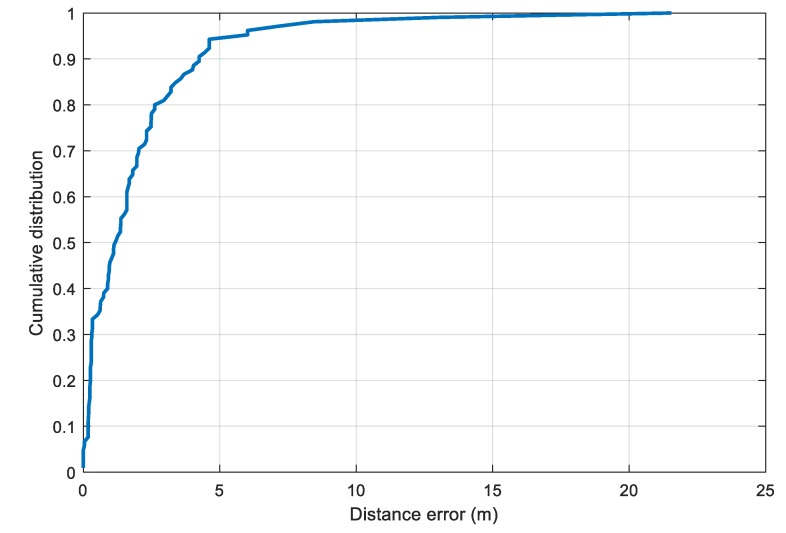
Cumulative distance errors with the selected parameters for the LDPL model in the obstacle-free setup.

To estimate the distance error, the two selected parameters $l_{0}$ and $rss_{l_{0}}$ are substituted into the model in Equation (10) to compute the distance from the RSS value. The cumulative distance error distribution is illustrated in Figure 9. From the plot, we set the value of $\delta_{b}$ in Equation (12) to 4.2 m, which corresponds to the 90th percentile of the distance error distribution.

### 5.3. Baseline Wi-Fi Fingerprinting Model

A Wi-Fi fingerprinting model is trained as a baseline model for the later fusion step with Bluetooth data. For building the Wi-Fi fingerprinting database, Device 1 (Samsung Galaxy Note 3) and Device 3 (Asus ME) are used. The database is built by using interpolation. First, one user is asked to carry the device and walk along the test path. The test path is predefined by several checkpoints (see Figure 7). Every time a checkpoint is reached, a timestamp is recorded and then used to interpolate the full moving path in a later stage. The database building process therefore does not take much time. There are six training log files in total. Given the specific testing path, all the data are collected in almost 1 h.

Table 3 gives an overview of the collected Wi-Fi database. A time threshold of 5 s is used to group RSS values into one complete fingerprint scan. There are around 400 fingerprints in six training files. The number of completed Wi-Fi scans per user’s walks varies between 50 and 100. Besides, the number of visible Wi-Fi access points also varies depending on the user location and the device in use. In our database, the average number of visible Wi-Fi access points per scan is almost 7, while the minimum is 3 and maximum is 12.

**Figure 10:**
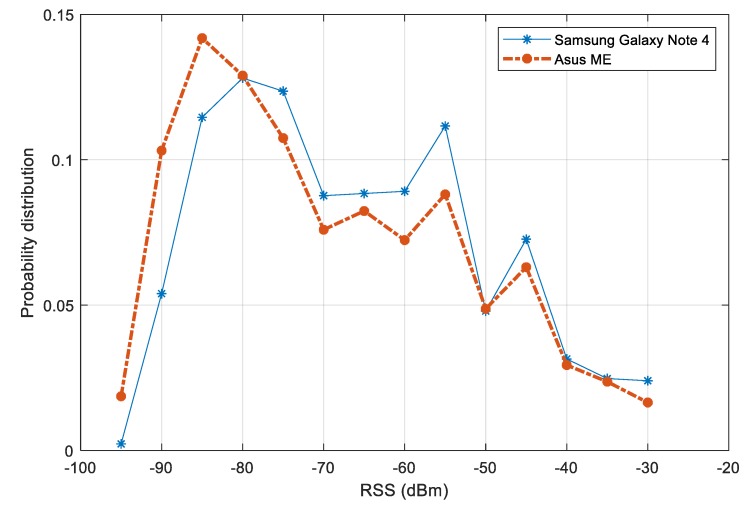
RSS value distribution of collected dataset for training fingerprinting model.

The distributions of RSS values from two devices is plotted Figure 10. A variation between the distributions of the two devices can be noticed. The variation is more significant in the range of signal strength from –90 dBm to –55 dBm. In a multiple-device context, this variation may affect the performance of fingerprinting models. To reduce the effect of device diversity, we first filter out the RSS values below –85 dBm. Second, a normalization step is carried out, so that for each participating device, it is assumed that the mean RSS value over the test area is known. The raw RSS values are then subtracted by this mean. Other alternative approaches such as finding the linear transformation or using HLF features would be suitable for improving the later step of fingerprinting model training.

For the fingerprinting model, we use Random Forest as the training model. The Random Forest is trained with 500 trees. Both the raw and the mean normalized RSS features are used in the K-fold cross validation testing with K=5. As the path contains two floors with some transient segments within stairs, the target position is treated as a 2.5D coordinate. For a triplet (x,y,z), the z component is normalized to receive only one of three values: 0, 0.5, and 1. The values 0 and 1 represent the points in the eighth and ninth floors, respectively, while the value 0.5 indicates that the user is somewhere on the stairs. The distance error between the target point $p_{\mathrm{target}}=\left(x_{\mathrm{target}},y_{\mathrm{target}},z_{\mathrm{target}}\right)$ and the output position p=(x,y,z) is calculated by:

(24) $$dist\left(p_{\mathrm{target}},p\right)=\sqrt{{\left(x_{\mathrm{target}}-x\right)}^{2}+{\left(y_{\mathrm{target}}-y\right)}^{2}}+k_{f}\times \left|z_{\mathrm{target}}-z\right|,$$

where $k_{f}$ is a floor multiplier. In our experiment, $k_{f}$ is set to 10.

**Figure 11:**
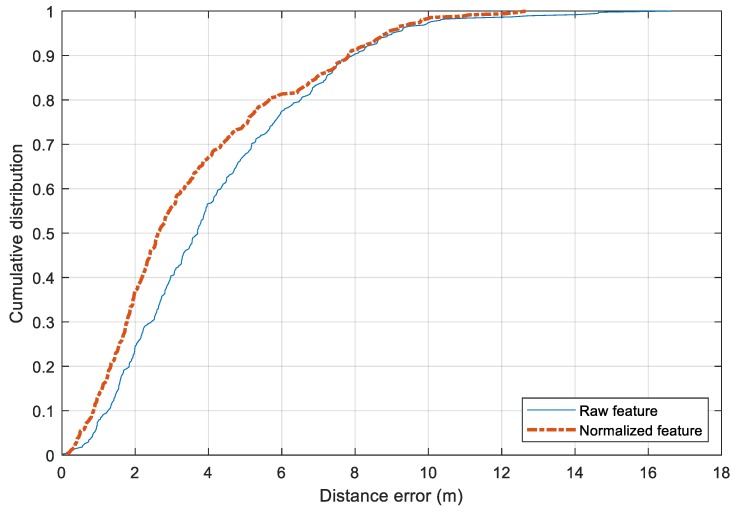
Comparison between the raw feature and the normalized feature in training with Random Forest model.

The performance of the two preprocessing feature approaches is shown in Figure 11. The standard raw features result in a mean distance error of 4.21 m while the mean normalized one has a mean error of 3.61 m. The former has a standard deviation of 2.65 m and the latter of 2.53 m. There is a slight improvement by changing from the raw features to the preprocessed one. In terms of maximum distance error, both approaches suffer from large errors that exceed 10 m. Compared with the published dataset of IPIN 2016 [1], the performance of the Random Forest model with the preprocessing features is comparable. To reduce the error further, it would require a larger amount of training data.

### 5.4. Evaluation Setup

Three approaches are used to localize the users: *Wi-Fi only*, *Non-temporal* and *Temporal*. In the *Wi-Fi only* approach, the output of the Wi-Fi fingerprinting method is provided as the reference tracking results to evaluate the performance of the two other approaches. First, the data is collected along the designed walking path. The baseline Random Forest (RF) regressor model is used without utilizing the Bluetooth data. In the testing phase, for each incoming Wi-Fi scan from the test devices, the RF model is used to estimate the user position.

In the *Non-temporal* approach, the output positions from the pre-trained RF regressor model on Wi-Fi are calculated for each device. If there is any Bluetooth data available, the Bluetooth scanned information is used for adjusting the positions of two involved devices. To solve the problem of non-simultaneous events between Wi-Fi and Bluetooth scans, we use a time window of length Δt of 10 s for grouping successive events into the same timestamp. The resulting position is calculated as the mean value of these positions.

In the *Temporal* approach, a RF classifier model is built on top of the RF regressor model. To transform the real-world coordinates to a label index, we perform K-means clustering of all the available training positions from the training data. The new learning targets are the indices of the corresponding clusters. In our experiments, we use K=30 for clustering all the available points in the tested area. The radius of each cluster in this configuration is 4.0 m approximately. The probability output of the classifier model is then used to update the Particle Filter within a time window of 10 s. If there are multiple completed scans in this time window, the closest completed scan is used. The Bluetooth data have the effective time set to 2.0 s. Regarding the moving model, the average speed of each particle is set to 1 m/s. In the simulation step, the number of particles is set to 1000.

### 5.5. Results and Discussion

**Figure 12:**
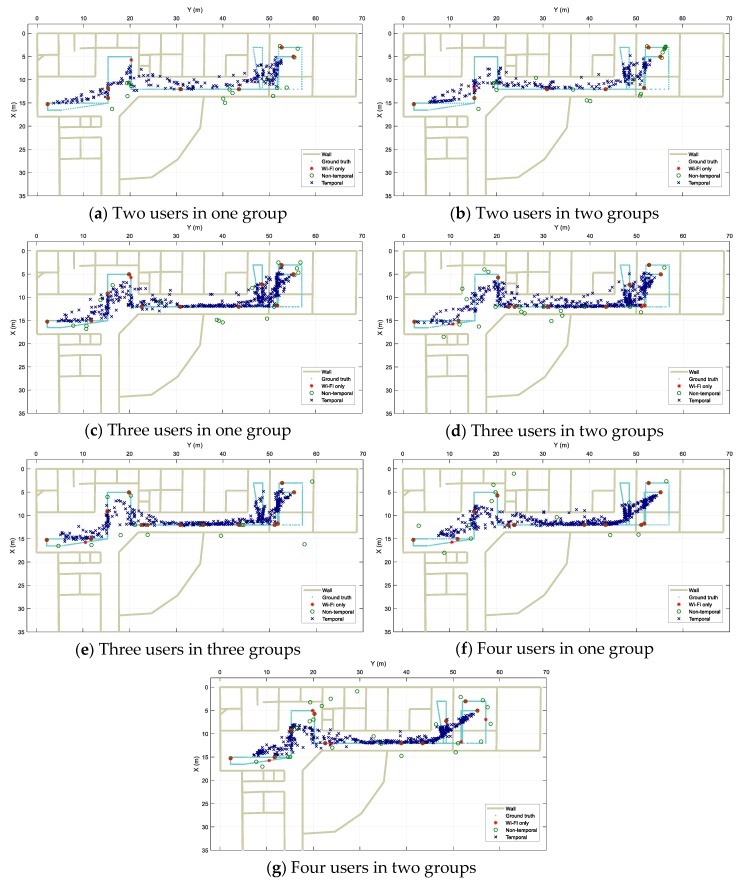
Localization results for designed scenarios. (**a**) Two users walking together in single group. (**b**) To users walking separately. (**c**) Three users walking all together in single group. (**d**) Three users walking where two are in group and another separately. (**e**) Three users walking separately. (**f**) Four users walking all together in single group. (**g**) Four users where each two walking together in group.

Figure 12 shows the localization output results along with the users’ walking ground truth from the three approaches for different scenarios. Visually, it can be observed that in all the experiments, *Wi-Fi only* performs very poorly with highly disjointed spots as multiple data scans result in fixed pre-trained locations due to the nature of the fingerprinting method in use. When fusion is applied, the Wi-Fi based results are combined with Bluetooth-based ones and spread along the real path. However, for the *Non-temporal* approach, because of the grouping of data using time windows, the resulting locations are much more disjointed in comparison with those from the *Temporal* approach. It is clear in the figure that the *Temporal* results track the real path more continuously.

A summary of the tracking results using the three mentioned approaches is presented in Table 4. In the configurations with only one user per group (i.e., number of users equals number of groups), the users are asked to move separately. In other configurations, users are asked to move with distance to each other kept under 10 m. The last scenario is with four users divided into two groups of two, which lets the system use Bluetooth data to identify both close and distant devices. The results are reported as the mean average distance errors across all the testing devices in the specific situation.

The *Wi-Fi only* approach reaches a stable performance of under 4.0 m in mean distance error. Both *Non-temporal* and *Temporal* approaches have better results than the *Wi-Fi only* one. However, the *Non-temporal* approach’s results are not as stable as the *Temporal* one. In the setup where the users’ distance could change within a specific time interval, the *Non-temporal* approach has a similar performance to the output from the Wi-Fi fingerprinting model. In this case, a difficulty is raised in measuring the distance in Equation (11). Meanwhile, the *Temporal* approach has a more consistent performance by reducing the errors from 25% to 50% depending on different scenarios. The biggest relative improvement is the scenario of three users moving in one group.

In the experiments with two users, Devices 1 and 3 are used, where the results for each approach per device are given in Table 5. In the first scenario, they walk together while keeping their distance under 1 m. In the other scenario, they walk separately and keep their distance in the range of 5–10 m. As both devices are also used in collecting the training data, the Wi-Fi fingerprinting model is unaffected by the device diversity in this case. When the information from the Bluetooth scanning process is added, the distance error is reduced in both *Non-temporal* and *Temporal* approaches. The *Non-temporal* has a clear improvement in the one-group scenario. However, the impact of Bluetooth data when discarding the temporal relationship is insignificant in the two-group scenario. With the *Temporal* approach, we have stable improvements across the two scenarios.

For experiments with three users, Devices 1, 3 and 4 are used, and the results are summarized in Table 6. With the *Wi-Fi only* approach, the RF regressor model results in similar distance errors for Devices 1 and 3. The positioning results for Device 4 are not as good as the other two devices because the data does not contain training samples for Device 4. Compared to the *Wi-Fi only* approach, there is a slight improvement by using the *Non-temporal* one. Across all the devices and scenarios, the average improvement is around 0.5 m in mean distance error. The appearance of Device 4 with high distance error clearly affects the performance of the other two devices. The *Temporal* approach provides more stable improvement compared to the *Wi-Fi only* by outperforming the two other approaches in all scenarios. The highest improvement is with Device 1 in one-group and Device 3 in three-group settings. With the contribution of the motion model and the Bluetooth-based distance, the *Temporal* approach could make the effect of non-training data on Device 4 minimal.

Results for four users are shown in Table 7. Compared to the previous one-group scenarios with two or three users, it is difficult to keep a close relative distance in this step. The group is supposed to move within a circle of 2 m in radius. In the second scenario with two groups, the first group uses Devices 1 and 2 whereas the second uses Devices 3 and 4. The distance between the two groups is kept in a range of 5–10 m. Similar results for the three-user case can be seen for the *Wi-Fi only* approach. Both new Devices 2 and 4 have larger distance errors than the two others. Devices 1 and 3 have a similar performance to the five-fold cross validation testing. It is noticeable that the RF fingerprinting model has trouble in tracking Devices 2 and 4, which are not present in the training data. High errors from RF fingerprinting model add more noise into the later fusion step with the Bluetooth information. The *Non-temporal* approach can improve the results in most cases. However, its impact is quite low. There are several exceptions where using the Bluetooth data makes the results worse. For example, the results from Device 1 increase from 3.05 m to 3.46 m in the one-group setting. The *Temporal* approach results in lower errors than the two others. In general, good results are obtained with Devices 1 and 3, whose RSS data are present in the training phase. There is an exception for the results of Device 2 in the one-group scenario, that is the *Temporal* mean error is slightly higher than that of the *Non-temporal*. Nevertheless, it is able to reduce to errors compared with the *Wi-Fi only* approach.

**Figure 13:**
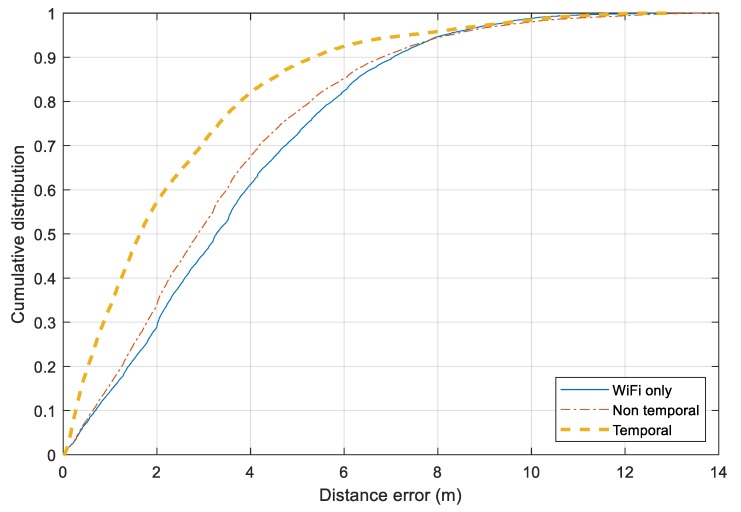
Cumulative distance error distribution for three approaches.

Figure 13 illustrates the distance error for three approaches over all the scenarios together. The *Wi-Fi only* and *Non-temporal* approaches perform similarly. For 75% of the time, the distance errors of two approaches are around 5 m. The Bluetooth-based relative distance is employed more efficiently in the *Temporal* approach. It has a significant improvement from the Wi-Fi-based tracking. For 75% and 90% of the time, the errors of the *Temporal* and *Non-temporal* approaches remain under 3.0 m and 5.0 m, respectively. Besides the Bluetooth information, employing map information and moving model constraints also help to reduce noisy output from the standard Wi-Fi fingerprinting model.

**Figure 14:**
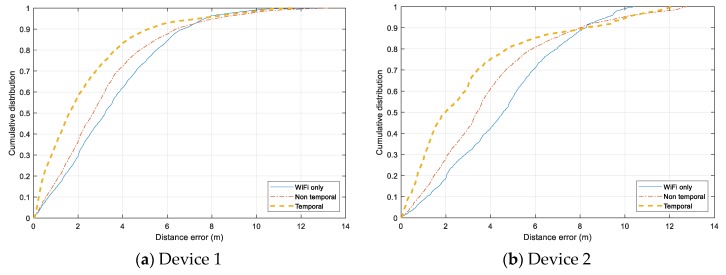

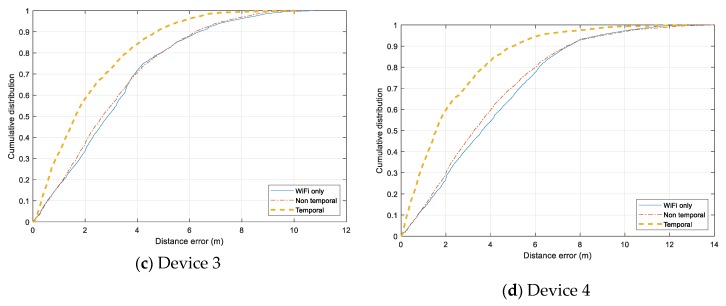
Cumulative distance error distribution for each testing device. (**a**) Samsung Galaxy Note 4 smartphone. (**b**) HTC One ME smartphone. (**c**) Asus ME tablet. (**d**) Samsung Galaxy Tab tablet.

Individual error distribution for each test device is given in Figure 14. Both smartphones, Samsung Galaxy Note 4 (Device 1) and HTC One ME (Device 2) have a similar distribution. The *Non-temporal* approach gains a slight improvement from using only Wi-Fi data, and the *Temporal* approach can reduce the error significantly for the range under 7.5 m. However, the additional Bluetooth data does not help when the tracking error exceed 8 m, and the error of all three models are distributed similarly in the range above 10 m. It even adds more noise to the tracking results of Device 2. In the case of Device 3 and Device 4, both *Non-temporal* and *Wi-Fi only* have nearly identical distributions while the *Temporal* one outperforms the two others. The *Temporal* has the highest improvement with Device 4, which can overcome the issue of non-training data.

## 6. Conclusions

In this work, a collaborative tracking framework based on the smartphone’s Wi-Fi and Bluetooth scanning data is introduced. The Wi-Fi data is used as a raw positioning output, which is then improved using the relative distance from Bluetooth inquiry RSS values. Two combination approaches are introduced, namely *Non-temporal* and *Temporal* approaches. The *Non-temporal* approach attempts to simplify the information fusion task by ignoring the time relationship between different Wi-Fi and Bluetooth scans. The *Temporal* approach takes a more direct way to establish the conditions between the two types of data. Both approaches have been tested and compared with a standard Wi-Fi fingerprinting model. From the testing results, while the *Non-temporal* is only applicable in some specific scenarios, the *Temporal* approach outperforms the Wi-Fi fingerprinting models significantly. Results show that the collaborative positioning based on the Wi-Fi and Bluetooth data would be applicable in a multi-user context. Combining two types of wireless data can reduce the noise from the Wi-Fi fingerprinting model significantly.

The testing scenario is still dealing with simple contexts of multiple users. There are also some remaining issues on the technical aspects, such as the communication between the users and the server, energy impacts on the smartphones, and signal inference between multiple devices. For the *Temporal*, we are currently using a simplistic particle filter based on distributions with constant parameters (e.g., moving speed, heading direction), which can be targeted for further improvement. Moreover, for demonstration of the effect gained by adding Bluetooth information, a simple Wi-Fi model is used in the experiments, but can be replaced by more advanced models in reality.

## Figures and Tables

**Table 1:** An example dataset with raw RSS feature for 3 access points.

Samples	$AP_{1}$	$AP_{2}$	$AP_{3}$
$X_{1}$	−50 dBm	−70 dBm	−75 dBm
$X_{2}$	−65 dBm	−80 dBm	−52 dBm
$X_{3}$	−70 dBm	−65 dBm	−76 dBm
$X_{1}$	−50 dBm	−70 dBm	−75 dBm

**Table 2:** Participating devices in the testing scenarios.

Device ID	Name	Type
1	Samsung Galaxy Note 4	Smartphone
2	HTC One ME	Smartphone
3	Asus ME	Tablet
4	Samsung Galaxy Tab	Tablet

**Table 3:** Data for training the Wi-Fi fingerprinting model.

Parameter	Value
Number of train files	6
Path length	220 m
Average time length	300 s
Average number of scans for one path	63
Total number of seen access points	138
Average number of seen access points	7

**Table 4:** Average positioning errors for all devices together in designed scenarios.

No. of Users	No. of Groups	Wi-Fi Only (m)	Non-Temporal (m)	Non-Temporal Improvement (%)	Temporal (m)	Temporal Improvement (%)
2	1	3.7 ± 2.0	2.4 ± 1.6	35.1	2.2 ± 1.5	40.5
2	2	3.4 ± 2.4	3.2 ± 2.3	5.9	2.5 ± 2.0	26.5
3	1	4.0 ± 2.4	3.5 ± 2.2	12.5	2.1 ± 1.8	47.5
3	2	3.4 ± 2.4	3.1 ± 2.2	9.8	2.4 ± 2.0	30.4
3	3	3.6 ± 2.1	3.1 ± 2.2	13.9	2.2 ± 1.9	38.9
4	1	3.8 ± 2.6	3.6 ± 2.6	5.3	2.8 ± 2.0	26.3
4	2	3.8 ± 2.4	3.5 ± 2.3	7.9	2.6 ± 2.1	31.6

**Table 5:** Average positioning errors per device for two-user experiments.

No. of Groups	Device ID	Wi-Fi Only (m)	Non-Temporal (m)	Temporal (m)
1	1	3.7 ± 2.1	2.6 ± 1.6	2.2 ± 1.6
1	3	3.7 ± 1.8	2.3 ± 1.6	2.2 ± 1.5
2	1	3.8 ± 2.8	3.9 ± 2.6	2.9 ± 2.2
2	3	3.1 ± 1.9	2.7 ± 1.9	2.1 ± 1.8

**Table 6:** Average positioning errors per device for three-user experiments.

No. of Groups	Device ID	Wi-Fi Only (m)	Non-Temporal (m)	Temporal (m)
1	1	3.8 ± 2.1	3.0 ± 2.2	2.2 ± 2.1
1	3	3.5 ± 2.3	3.3 ± 2.1	2.0 ± 1.7
1	4	4.8 ± 2.9	4.3 ± 2.2	2.2 ± 1.7
2	1	3.0 ± 2.1	2.8 ± 1.9	2.3 ± 1.7
2	3	3.4 ± 2.6	3.3 ± 2.3	2.8 ± 2.4
2	4	3.9 ± 2.6	3.3 ± 2.3	2.1 ± 1.8
3	1	3.3 ± 2.4	3.0 ± 2.4	2.2 ± 1.9
3	3	3.5 ± 2.0	2.7 ± 1.9	2.1 ± 1.7
3	4	3.9 ± 2.0	3.8 ± 2.4	2.4 ± 2.0

**Table 7:** Average positioning errors per device for four-user experiments.

No. of Groups	Device ID	Wi-Fi Only (m)	Non-Temporal (m)	Temporal (m)
1	1	3.1 ± 1.9	3.5 ± 2.3	1.9 ± 1.5
1	3	5.0 ± 3.6	4.3 ± 3.2	4.6 ± 3.1
1	4	3.5 ± 2.6	3.0 ± 2.3	2.4 ± 1.8
1	1	3.5 ± 2.6	3.3 ± 2.4	2.3 ± 1.6
2	3	3.2 ± 2.3	2.6 ± 1.7	2.7 ± 2.0
2	4	4.2 ± 2.5	3.5 ± 2.2	3.2 ± 2.7
2	1	3.5 ± 2.2	3.9 ± 2.3	1.7 ± 1.6
2	3	4.2 ± 2.7	3.9 ± 2.9	2.8 ± 2.5
